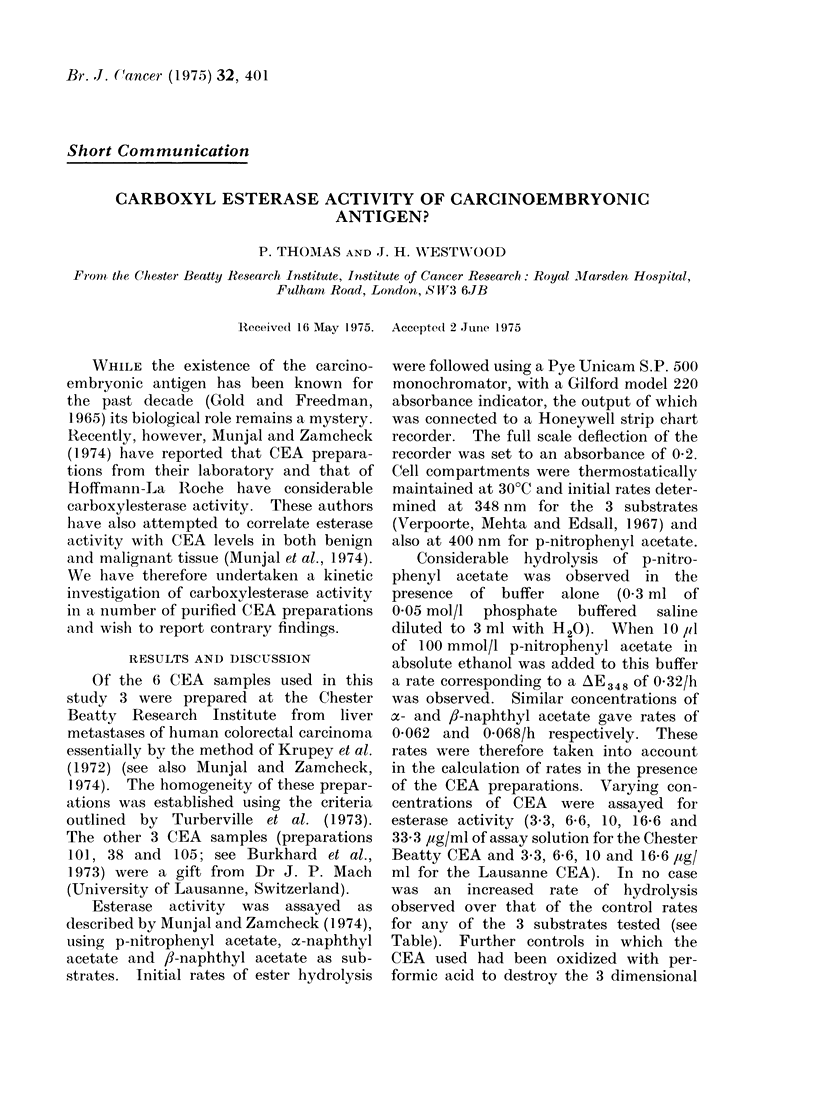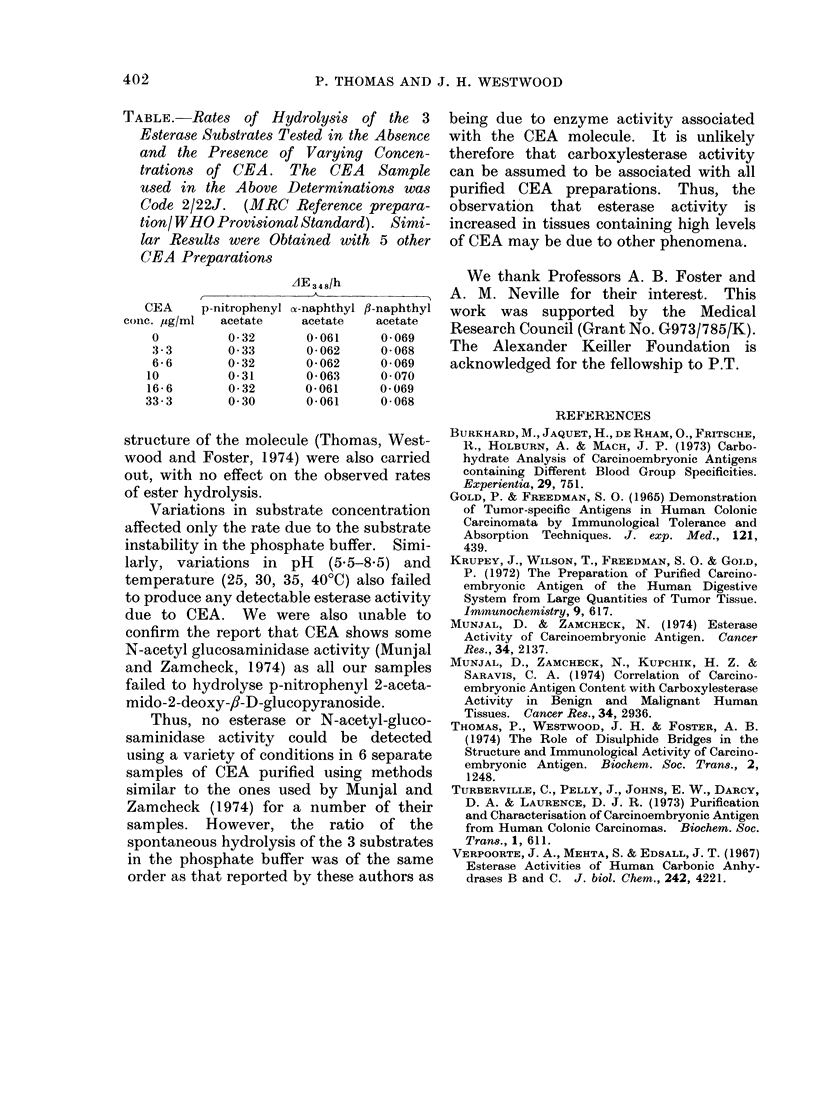# Carboxyl esterase activity of carcinoembryonic antigen?

**DOI:** 10.1038/bjc.1975.240

**Published:** 1975-09

**Authors:** P. Thomas, J. H. Westwood


*Br. J. Cancer* (1975) **32**, 401

***Short Communication***

### CARBOXYL ESTERASE ACTIVITY OF CARCINOEMBRYONIC ANTIGEN?

P. THOMAS AND J. H. WESTWOOD

*From the Chester Beatty Research Institute, Institute of Cancer Research: Royal Marsden Hospital, Fulham Road, London, SW3 6JB*

Received 16 May 1975. Accepted 2 June 1975

WHILE the existence of the carcinoembryonic antigen has been known for the past decade (Gold and Freedman, 1965) its biological role remains a mystery. Recently, however, Munjal and Zamcheck (1974) have reported that CEA preparations from their laboratory and that of Hoffmann-La Roche have considerable carboxylesterase activity. These authors have also attempted to correlate esterase activity with CEA levels in both benign and malignant tissue (Munjal *et al.*, 1974). We have therefore undertaken a kinetic investigation of carboxylesterase activity in a number of purified CEA preparations and wish to report contrary findings.

### RESULTS AND DISCUSSION

Of the 6 CEA samples used in this study 3 were prepared at the Chester Beatty Research Institute from liver metastases of human colorectal carcinoma essentially by the method of Krupey *et al.* (1972) (see also Munjal and Zamcheck, 1974). The homogeneity of these preparations was established using the criteria outlined by Turberville *et al.* (1973). The other 3 CEA samples (preparations 101, 38 and 105; see Burkhard *et al.*, 1973) were a gift from Dr J. P. Mach (University of Lausanne, Switzerland).

Esterase activity was assayed as described by Munjal and Zamcheck (1974), using p-nitrophenyl acetate, α-naphthyl acetate and β-naphthyl acetate as substrates. Initial rates of ester hydrolysis were followed using a Pye Unicam S.P. 500 monochromator, with a Gilford model 220 absorbance indicator, the output of which was connected to a Honeywell strip chart recorder. The full scale deflection of the recorder was set to an absorbance of 0·2. Cell compartments were thermostatically maintained at 30°C and initial rates determined at 348 nm for the 3 substrates (Verpoorte, Mehta and Edsall, 1967) and also at 400 nm for p-nitrophenyl acetate.

Considerable hydrolysis of p-nitrophenyl acetate was observed in the presence of buffer alone (0·3 ml of 0·05 mol/l phosphate buffered saline diluted to 3 ml with $H_2O$). When 10 μl of 100 mmol/l p-nitrophenyl acetate in absolute ethanol was added to this buffer a rate corresponding to a $\Delta E_{348}$ of 0·32/h was observed. Similar concentrations of α- and β-naphthyl acetate gave rates of 0·062 and 0·068/h respectively. These rates were therefore taken into account in the calculation of rates in the presence of the CEA preparations. Varying concentrations of CEA were assayed for esterase activity (3·3, 6·6, 10, 16·6 and 33·3 μg/ml of assay solution for the Chester Beatty CEA and 3·3, 6·6, 10 and 16·6 μg/ml for the Lausanne CEA). In no case was an increased rate of hydrolysis observed over that of the control rates for any of the 3 substrates tested (see Table). Further controls in which the CEA used had been oxidized with performic acid to destroy the 3 dimensional

TABLE.—*Rates of Hydrolysis of the 3 Esterase Substrates Tested in the Absence and the Presence of Varying Concentrations of CEA. The CEA Sample used in the Above Determinations was Code 2/22J. (MRC Reference preparation/WHO Provisional Standard). Similar Results were Obtained with 5 other CEA Preparations*

| CEA conc. μg/ml | $\Delta E_{348}$/h p-nitrophenyl acetate | α-naphthyl acetate | β-naphthyl acetate |
|---|---|---|---|
| 0 | 0·32 | 0·061 | 0·069 |
| 3·3 | 0·33 | 0·062 | 0·068 |
| 6·6 | 0·32 | 0·062 | 0·069 |
| 10 | 0·31 | 0·063 | 0·070 |
| 16·6 | 0·32 | 0·061 | 0·069 |
| 33·3 | 0·30 | 0·061 | 0·068 |

structure of the molecule (Thomas, Westwood and Foster, 1974) were also carried out, with no effect on the observed rates of ester hydrolysis.

Variations in substrate concentration affected only the rate due to the substrate instability in the phosphate buffer. Similarly, variations in pH (5·5–8·5) and temperature (25, 30, 35, 40°C) also failed to produce any detectable esterase activity due to CEA. We were also unable to confirm the report that CEA shows some N-acetyl glucosaminidase activity (Munjal and Zamcheck, 1974) as all our samples failed to hydrolyse p-nitrophenyl 2-acetamido-2-deoxy-β-D-glucopyranoside.

Thus, no esterase or N-acetyl-glucosaminidase activity could be detected using a variety of conditions in 6 separate samples of CEA purified using methods similar to the ones used by Munjal and Zamcheck (1974) for a number of their samples. However, the ratio of the spontaneous hydrolysis of the 3 substrates in the phosphate buffer was of the same order as that reported by these authors as being due to enzyme activity associated with the CEA molecule. It is unlikely therefore that carboxylesterase activity can be assumed to be associated with all purified CEA preparations. Thus, the observation that esterase activity is increased in tissues containing high levels of CEA may be due to other phenomena.

We thank Professors A. B. Foster and A. M. Neville for their interest. This work was supported by the Medical Research Council (Grant No. G973/785/K). The Alexander Keiller Foundation is acknowledged for the fellowship to P.T.

### REFERENCES

BURKHARD, M., JAQUET, H., DE RHAM, O., FRITSCHE, R., HOLBURN, A. & MACH, J. P. (1973) Carbohydrate Analysis of Carcinoembryonic Antigens containing Different Blood Group Specificities. *Experientia*, **29**, 751.

GOLD, P. & FREEDMAN, S. O. (1965) Demonstration of Tumor-specific Antigens in Human Colonic Carcinomata by Immunological Tolerance and Absorption Techniques. *J. exp. Med.*, **121**, 439.

KRUPEY, J., WILSON, T., FREEDMAN, S. O. & GOLD, P. (1972) The Preparation of Purified Carcinoembryonic Antigen of the Human Digestive System from Large Quantities of Tumor Tissue. *Immunochemistry*, **9**, 617.

MUNJAL, D. & ZAMCHECK, N. (1974) Esterase Activity of Carcinoembryonic Antigen. *Cancer Res.*, **34**, 2137.

MUNJAL, D., ZAMCHECK, N., KUPCHIK, H. Z. & SARAVIS, C. A. (1974) Correlation of Carcinoembryonic Antigen Content with Carboxylesterase Activity in Benign and Malignant Human Tissues. *Cancer Res.*, **34**, 2936.

THOMAS, P., WESTWOOD, J. H. & FOSTER, A. B. (1974) The Role of Disulphide Bridges in the Structure and Immunological Activity of Carcinoembryonic Antigen. *Biochem. Soc. Trans.*, **2**, 1248.

TURBERVILLE, C., PELLY, J., JOHNS, E. W., DARCY, D. A. & LAURENCE, D. J. R. (1973) Purification and Characterisation of Carcinoembryonic Antigen from Human Colonic Carcinomas. *Biochem. Soc. Trans.*, **1**, 611.

VERPOORTE, J. A., MEHTA, S. & EDSALL, J. T. (1967) Esterase Activities of Human Carbonic Anhydrases B and C. *J. biol. Chem.*, **242**, 4221.